# Population structure and selection signal analysis of indigenous sheep from the southern edge of the Taklamakan Desert

**DOI:** 10.1186/s12864-024-10581-y

**Published:** 2024-07-09

**Authors:** Zhi-peng Han, Rui-zhi Yang, Wen Zhou, Lu-lu Zhang, Jie-ru Wang, Chun-jie Liu, Shu-dong Liu

**Affiliations:** 1 College of Animal Science and Technology, Tarim University, Alar, 843300 China; 2 Key Laboratory of Tarim Animal Husbandry Science and Technology, Xinjiang Production and Construction Corps, Alar, 843300 China

**Keywords:** Taklamakan desert, Native sheep, Population structure, Selection signals, Adaptability

## Abstract

Analyzing the genetic diversity and selection characteristics of sheep (Ovis aries) holds significant value in understanding their environmental adaptability, enhancing breeding efficiency, and achieving effective conservation and rational utilization of genetic resources. In this study, we utilized Illumina Ovine SNP 50 K BeadChip data from four indigenous sheep breeds from the southern margin of the Taklamakan Desert (Duolang sheep: *n* = 36, Hetian sheep: *n* = 74, Kunlun sheep: *n* = 27, Qira black sheep: *n* = 178) and three foreign meat sheep breeds (Poll Dorset sheep: *n* = 105, Suffolk sheep: *n* = 153, Texel sheep: *n* = 150) to investigate the population structure, genetic diversity, and genomic signals of positive selection within the indigenous sheep. According to the Principal component analysis (PCA), the Neighbor-Joining tree (NJ tree), and Admixture, we revealed distinct clustering patterns of these seven sheep breeds based on their geographical distribution. Then used Cross Population Extended Haplotype Homozygosity (XP-EHH), Fixation Index (F_ST_), and Integrated Haplotype Score (iHS), we identified a collective set of 32 overlapping genes under positive selection across four indigenous sheep breeds. These genes are associated with wool follicle development and wool traits, desert environmental adaptability, disease resistance, reproduction, and high-altitude adaptability. This study reveals the population structure and genomic selection characteristics in the extreme desert environments of native sheep breeds from the southern edge of the Taklimakan Desert, providing new insights into the conservation and sustainable use of indigenous sheep genetic resources in extreme environments. Additionally, these findings offer valuable genetic resources for sheep and other mammals to adapt to global climate change.

**Supplementary Information:**

The online version contains supplementary material available at 10.1186/s12864-024-10581-y.

## Introduction

Sheep (Ovis aries) play a crucial role in providing various products for humans, including wool, skin, meat, and milk, making them one of the most economically significant agricultural species [1]. Domesticated approximately 11,000 years ago, Sheep have spread to different continents and adapted to diverse agricultural ecological conditions accompanied human migration [2], including extreme weather such as highlands and deserts. The Taklamakan Desert, located in the Tarim Basin within the Eurasian hinterland [3], features gravel deserts surrounded by several mountains, forming a ‘C’ type mountain-basin pattern [4]. The average annual precipitation in this basin is less than 50 millimeters, while evaporation averages around 2600 millimeters [5]. The region experiences frequent windy and dusty weather [6], large diurnal temperature variations, and abundant sunlight, typical of a temperate continental desert climate. The extreme environmental conditions pose significant challenges to livestock production [7]. Studying how domesticated animals adapt to extreme environments in a short period of time can help formulate appropriate breeding plans to mitigate the impact of the future environmental changes.

Natural selection plays a crucial role in shaping individuals better adapted to new environments. In addition to natural selection, artificial selection has been extensively implemented in livestock breeding to cultivate more desirable and advantageous individuals [8]. Notably, compared to traditional artificial selection methods, genomic selection expedites the process of identifying superior genotypes and accelerating the breeding cycle [9]. The employment of the Illumina Ovine SNP 50 K BeadChip in conjunction with selective trait detection not only uncovers the underlying mechanisms of modern breeding and artificial selection but also provide possibilities to identify the candidate genes associated with adaptively and economically important traits [10, 11]. In past studies of ruminants by others, a number of genes that directly cause trait variation when subjected to positive selection have been identified. Lei et al. [12]. analyzed 27 different populations of sheep with varying wool types based on the Illumina Ovine SNP 50 K Genotyping BeadChip. They identified four potential genes (*PRX*, *SOX18*, *TGM3*, and *TCF3*) directly or indirectly influencing wool follicle development and wool shedding. Cheng et al. [13]. analyzed the whole-genome sequencing data from 1167 sheep (1098 domestic sheep and 69 wild sheep) among the 161 breeds (154 breeds and 7 Ovis species). They highlighted the haplotypes closely associated with spiral horn characteristics (*RXFP2*), ear morphology (*MSRB3*), and facial features (*VPS13B*). Cao et al. [14]. used whole-genome single nucleotide polymorphism (SNP) data from 111 populations of domestic sheep (*n* = 3447) and 7 populations of wild sheep (*n* = 403) to detect clear signals of introgression from the wild relatives into the domestic populations. Ultimately, they identified signals of introgression associated with olfaction (*ADCY3*, *TRPV1*) and innate immunity (*PADI2*). Zhang et al. [15] examined the selection signatures of 5 sheep breeds with three different agricultural geographic characteristics based on the Illumina Ovine SNP 50 K Genotyping BeadChip. They found genetic information associated with immunity (*DNTT*, *FEN1*, *POLL*, *PRKDC*, *XRCC4*), high reproductive rate (*IGFBP7*, *STC1*, *TFAP2*), and adaptability (*PNPLA6*, *MITF*) in indigenous sheep breeds adapted to the Taklamakan desert environment using four complementary genomic selection signals (FST, XP-EHH, Rsb, and iHS). Kim et al. [16]. compared the genetic variation patterns of five indigenous African cattle populations from regions south of the Sahara with those of 53 commercial taurine breeds using the Illumina Bovine SNP 50 K Genotyping BeadChip. They revealed the genetic variations in African indigenous cattle related to gene selection characteristics associated with heat tolerance (*BTA22*, *HSPA4*, *SOD1*, *PRLH*), disease resistance (*HCRTR1*, *BOLA-DRB3*), coat color (*MC1R*, *KIT*, *MITF*, *PDGFRA*), and horn development (*MAP3K5*, *PPP2R2C*, *FGF18*, *FRS3*, *ACVRL1*, *CASR*, *TLX3*, *ACVR1B*, *RUNX3*).

Although the genetic basis underlying the important economic traits such as reproduction and year-round estrus in the indigenous sheep from the southern edge of the Taklamakan Desert has been reported [17], our understanding of their interpopulation genetic structure and the genetic mechanisms relating to their adaptation to the local environment is currently limited. This study aims to employ high-density 50 K SNP genetic data to uncover the population structure of the indigenous sheep from the southern edge of the Taklamakan Desert and assess the genomic signatures indicative of selective pressures in the extreme environmental conditions. Of greater significance, this investigation promises to offer new insights into the conservation and sustainable utilization of local sheep breeds’ genetic resources in the extreme desert environment. Additionally, it has the potential to deliver valuable resources for other mammals to cope with global climate change.

## Materials and methods

### Ethics statement

This work was conducted in accordance with the standards set by the Ethics Committee of the College of Animal Science and Technology of Tarim University (SYXK2020-009). Written informed consent was obtained from the owners for the participation of their animals in this study.

### Animal samples

**Fig. 1:**
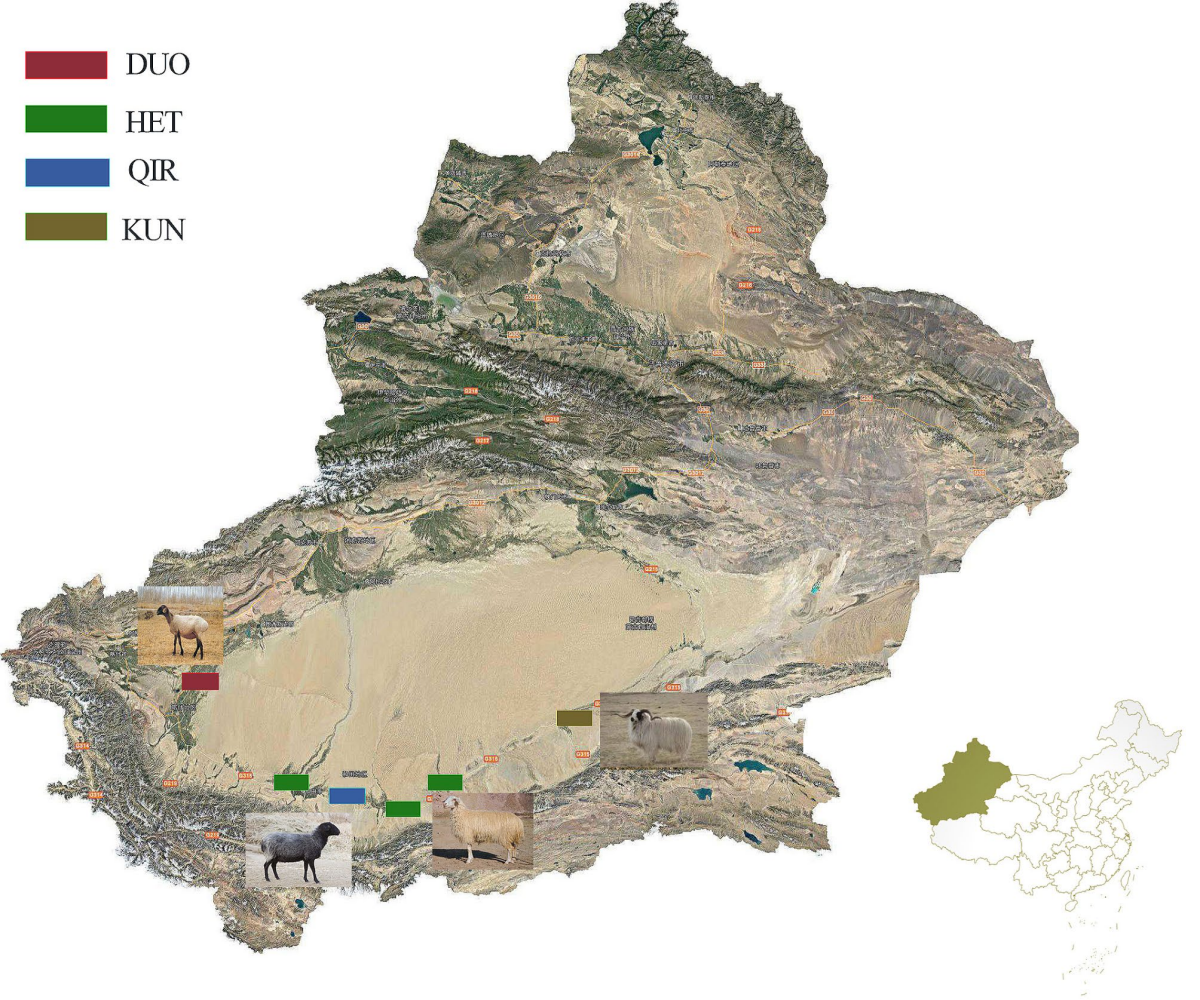
Map showing the different geographic distribution of the four indigenous sheep breeds from the southern edge of the taklamakan desert, china, analyzed in this study

In this study, a total of 723 samples from seven different breeds were included (Fig. 1; Table 1). To be specific, we used four native sheep breeds inhabiting the southern border of the Taklamakan Desert, including 36 Duo-lang sheep (DUO) from the Duo-lang Sheep Breeding Farm in Maigaiti County, Xinjiang; 178 Qira black sheep (QIR) from the Qira Black Sheep Breeding Farm in Cele County, Xinjiang; 74 Hetian sheep (HET) from Hetian District in Xinjiang; and 27 Kunlun sheep (KUN) from the Kunlun Sheep Breeding Farm in Qiemo County, Xinjiang. Samples were selected on a random sampling basis from different breeding groups, different pens, and different batches to ensure their unrelatedness. All samples underwent assessments based on factors such as dental wear, size development, and developmental stage to initially determine their age. Subsequently, the age data collected were then cross-referenced in detail with on-farm management records to verify the accuracy of the animals’ age. Female specimens were deliberately chosen to minimize any potential confounding effects stemming from sexual disparities. Furthermore, we obtained data of Poll Dorset sheep (POL, 105), Suffolk sheep (SUF, 153), and Texel sheep (TEX, 150) from the International Sheep Genomics Consortium (ISGC) 1.

**Table 1:** Information on the location of experimental animals and genomic diversity indices

Breed	Code	Location	Number of sample	Purpose	Elevation (m)	Inbreeding coefficient (F)	Minor allele frequency (MAF)	Observed heterozygosity (Ho)	Expected heterozygosity (He)
Duolang	DUO	Makati County, Xinjiang	36	Wool/Meat	1200	0.0116	0.2780	0.6264	0.6360
Qira Black	QIR	Celle County, Xinjiang	178	Lamb skin	1336	0.0222	0.2714	0.6450	0.6371
Hetian	HET	Moyu County, Xinjiang	29	Carpet wool	1100~1325	0.0464	0.2977	0.6219	0.6034
		Yutian County, Xinjiang	13		1460~2500				
		Minfeng County, Xinjiang	32		3000~4000				
Kunlun	KUN	Qiemo County, Xinjiang	27	Wool/Meat	1170~4500	-0.0746	0.2665	0.5898	0.6158
Poll Dorset	POL	Australia	105	Meat	272	-0.0172	0.2676	0.6259	0.6318
Suffolk	SUF	Australia	99	Meat	272	0.0396	0.2894	0.6366	0.6218
		Ireland	54		118				
Texel	TEX	Germany	46	Meat	271	0.0054	0.2775	0.6366	0.6347
		New Zealand	24		388				
		Scotland	80		162				

### Genotyping and quality control

Blood samples were collected from the jugular vein, and DNA was extracted using a DNA extraction kit (Tiangen Biotech Co Ltd., Beijing, China). DNA samples were genotyped using the Illumina Ovine SNP50K BeadChip (Compass Biotechnology Co Ltd., Beijing, China). The quality of the single nucleotide polymorphism genotyping data was evaluated using Plink V.1.90 software [18], and unqualified SNPs were excluded. The quality control criteria for this study were as follows: (1) individual detection rate > 0.95; (2) SNPs detection rate > 0.95; (3) Minor allele frequency (MAF) > 0.05; (4) Hardy-Weinberg equilibrium (HWE) p-value ≥ 1 × 10^− 6^.

### Genetic diversity

The inbreeding coefficient (F), observed heterozygosity (Ho), expected heterozygosity (He), and minor allele frequency (MAF) were calculated for the seven sheep breeds using Plink V. 1.90 software.

### Population structure analyses

After completing quality control, the genotypic data was subjected PCA analysis using Plink V. 1.90 software. The P distance matrix was calculated using VCF2Dis V. 1.09 software 2, which was then used to construct an NJ-tree via the ATGC: Montpellier Bioinformatics platform 3. Lastly, the NJ-tree was visualized using iTOL 4 online tool [19]. Genetic admixture calculations were executed using ADMIXTURE 1.3.0 [20], and the population genetic structure was analyzed by using K values ranging from 2 to 10. Additionally, respective cross-validation (CV) errors were calculated.

### Genome-wide selective signatures

To accurately reveal the genetic imprints left by indigenous sheep during their evolution at the southern edge of the Taklamakan Desert, we utilized several selection signal detection methods [21, 22]. Initially, we identified candidate gene regions in indigenous sheep that may have undergone strong natural selection utilizing F_ST_ and XP-EHH methods. Subsequently, we conducted an in-population genome scan on each of the four local sheep breeds using iHS. Finally, by cross-comparing the results obtained from F_ST_ and XP-EHH with the iHS scans, we identified positively selected alleles in each local sheep breed.

XP-EHH statistic is designed to detect ongoing or nearly fixed selection features by comparing haplotypes between two populations [23]. The formula is as follows:

$$XPEHH=\frac{In\left(\frac{I}{{I}_{i}}\right)-{E}_{p}[ln\left(\frac{I}{{I}_{i}}\right)]}{S{D}_{p}\left[\left(\frac{I}{{I}_{i}}\right)\right]}$$

XP-EHH values were calculated using Selscan v1.3.0 [24]. Positive values indicate selection signals in population I, while negative values indicate selection signals in population I_i_.

Fst is a measure that quantifies the degree of genetic differentiation between populations, making it suitable for comparing diversity among different subpopulations of the same species. The formula is as follows:

$${F}_{ST}=\frac{MSP-MSG}{MSP+({n}_{c}-1)MSG}$$

MSG represents the mean squared error within populations, MSP represents the mean squared error between populations, and n_c_ is the average sample size corrected for the entire population. Vcftools was used to calculate the Fst values for each window (window size of 50 Kb, sliding step size of 25 Kb). The thresholds of 1% of these two selective signatures were considered as potential candidate regions under selection.

IHS is a statistic based on haplotype frequency to detect selection signals within the genome [25]. The formula is as follows:

$$iHS={ln}\left(\frac{{iHH}_{A}}{{iHH}_{D}}\right)$$

IHS denotes the integrated haplotype value, iHHA denotes the EHH integration score of the ancestral allele, and iHHD denotes the EHH integration score of the derived allele. The obtained iHS values were corrected using Voight’s method with the following formula:

$$niHS=\frac{{ln}\left(\frac{{iHH}_{A}}{{iHH}_{D}}\right)-E\left[{ln}\left(\frac{{ihh}_{A}}{{iHH}_{D}}\right)\right]}{SD\left[{ln}\left(\frac{{iHH}_{A}}{{iHH}_{D}}\right)\right]}$$

$$E\left[{ln}\left(\frac{{ihh}_{A}}{{iHH}_{D}}\right)\right]$$

represents the expected iHS value, and

$$SD\left[{ln}\left(\frac{{iHH}_{A}}{{iHH}_{D}}\right)\right]$$

represents the standard deviation. A large iHS value suggests that the haplotype likely carries ancestral alleles, whereas a small iHS value indicates the likely possess of evolved genes. Candidate regions under selection were determined based on the 5% threshold of iHS.

### Enrichment analysis of candidate genes

Gene annotation was performed utilizing the sheep genome Oar_v4.0. Gene functional annotation was conducted referencing the NCBI 5 and OMIM 6 databases. Gene clustering analysis was carried out using DAVID 7. Pathway enrichment analysis was performed utilizing Gene Ontology 8(GO) and Kyoto Encyclopedia of Genes and Genomes 9(KEGG). Performed gene networking analysis on the overlapping candidate genes obtained through paired comparisons.

## Results

### SNP quality control

In the experiment, a total of 46,900 SNPs from 723 sheep underwent genotypic quality control. After removing SNPs that did not meet the quality control criteria, 41,831 SNPs remained for further analysis.

### Genetic diversity

The genetic diversity results for the seven sheep breeds are as follows (Table 1):

KUN has the lowest F (-0.0746), while HET has the highest (0.0464).

KUN has the lowest MAF (0.2665), while HET has the highest (0.2977).

KUN has the lowest Ho (0.5898), while QIR has the highest (0.6450).

HET has the lowest He (0.6034), while QIR has the highest (0.6371).

### Population genetic structure

We utilized PCA, NJ tree, and ADMIXTURE methods to analyze the genetic relationships and structure among four native sheep breeds from the southern edge of the Taklamakan Desert and three sheep breeds from other countries.

**Fig. 2:**
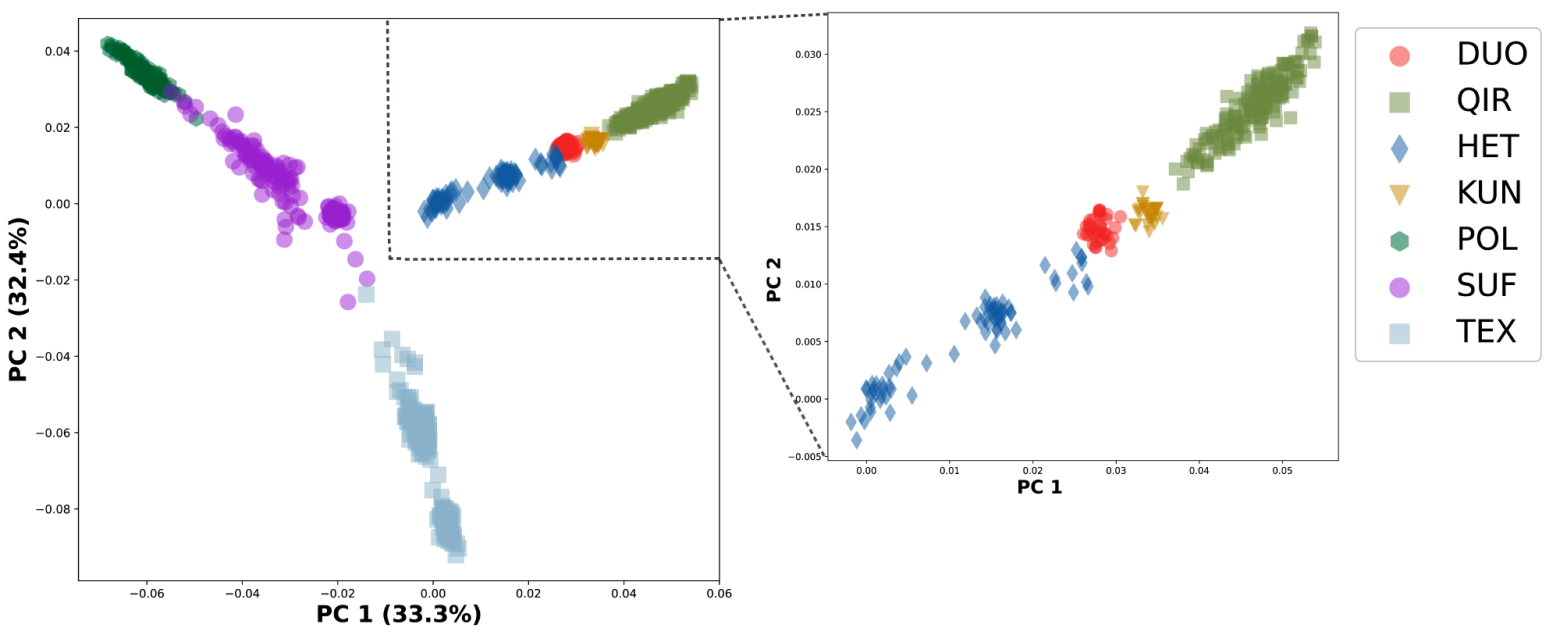
The PCA analysis chart of sheep breeds from two different geographical regions, the X-axis represents PC1, and the Y-axis is PC2. The picture on the left shows the genetic distances of seven sheep breeds, and that on the right shows the genetic distances of four sheep breeds from the southern edge of the taklimakan desert

**Fig. 3:**
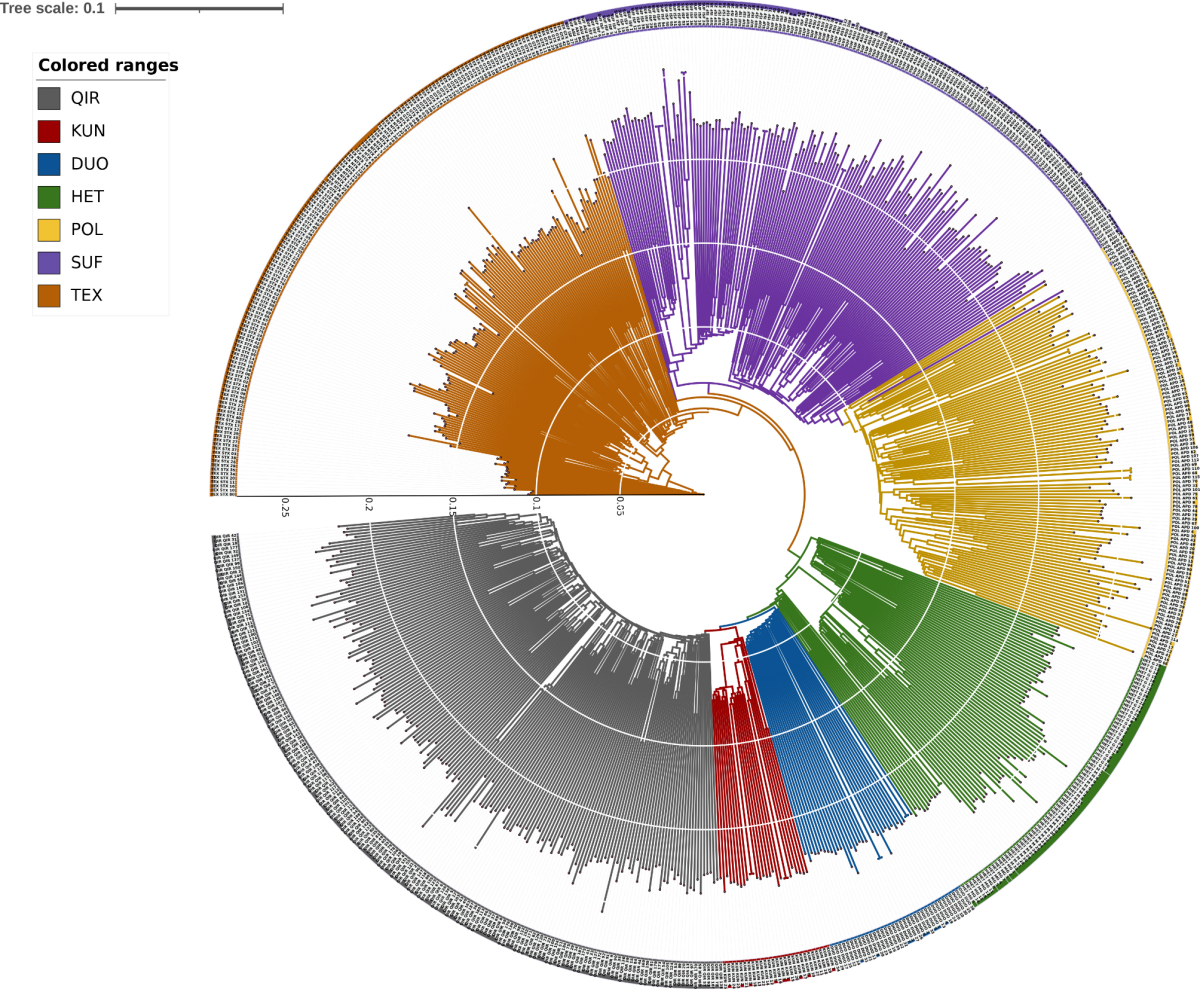
Evolutionary tree of seven sheep breeds. Gray represents QIR, Red represents KUN, Blue represents DUO, Green represents HET, Yellow represents POL, Purple represents SUF, and Brown represents TEX

**Fig. 4:**
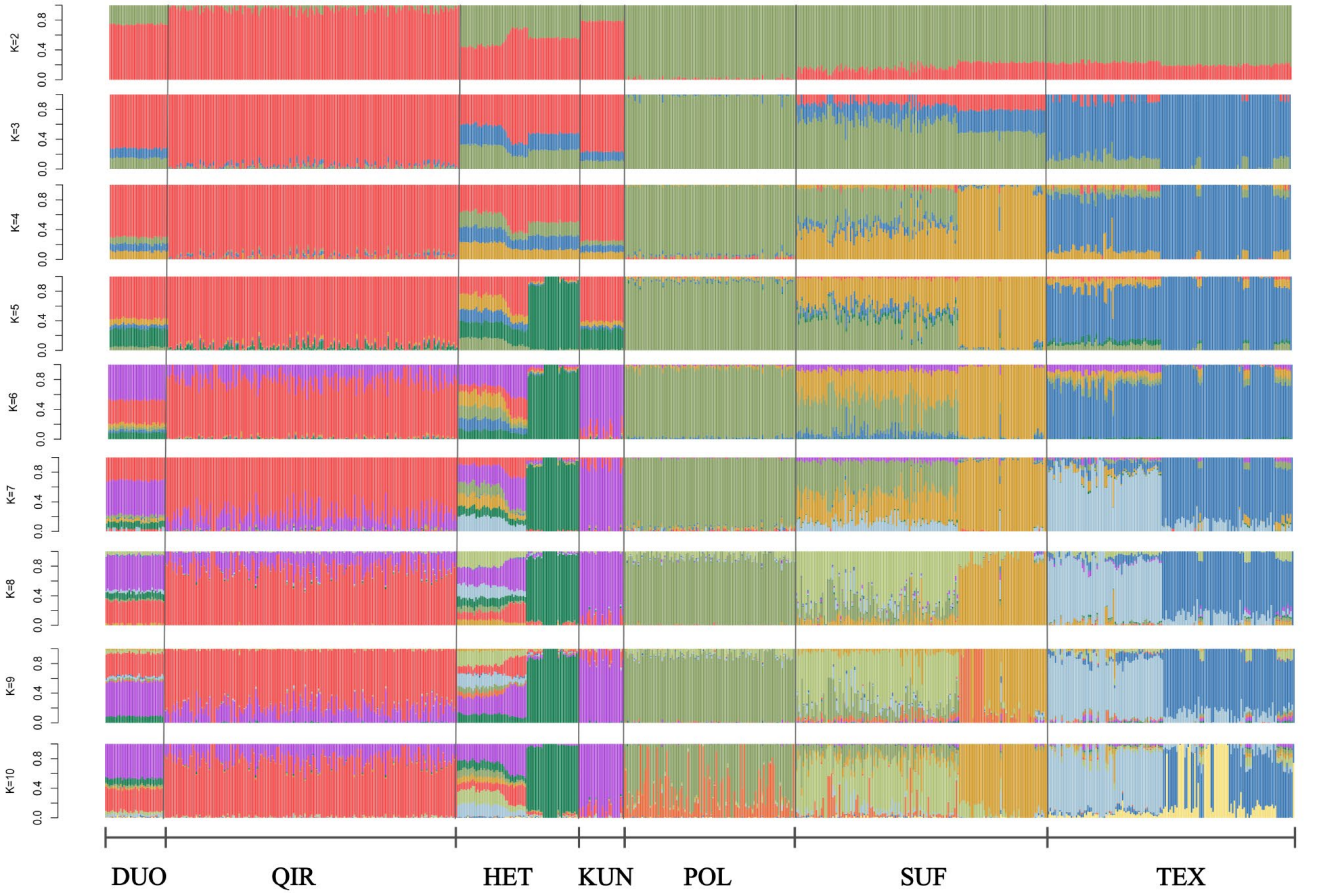
Admixture analysis of seven sheep breeds. Results for inferred numbers of clusters k = 2–10 were shown. Different colors represent different ancestral components. From left to right are DUO, QIR, HET, KUN, POL, SUF and TEX

In the PCA results (Fig. 2) revealed a distinct separation between the sheep breeds from other countries (POL, SUF, TEX) and the native sheep breeds from the southern edge of the Taklamakan Desert (DUO, QIR, KUN, HET). Furthermore, the native sheep breeds from the southern edge of the Taklamakan Desert exhibited further differentiation based on their geographic distribution, with HET appearing to be divided into three subgroups. The NJ tree accurately differentiated the seven sheep breeds based on their geographic origins (Fig. 3). The native sheep breeds from the southern edge of the Taklamakan Desert coalesced into one branch, whereas the sheep breeds from other countries constituted the other branch. Notably, the NJ tree revealed that HET was subdivided into three clusters, with one cluster aligning with DUO, QIR, and KUN, while the other two clusters formed separate groups. The results closely align with those obtained from the PCA analysis. The ADMIXTURE results showed (Fig. 4) that the sheep breeds from other countries and the native sheep from the southern edge of the Taklamakan Desert possess different ancestral components. At K = 4, QIR predominantly belonged to the genomic cluster specific to the native sheep breeds from the southern edge of the Taklamakan Desert. HET showed the highest level of admixture, with no dominant genomic cluster among the four clusters. At K = 5, a new genomic cluster emerged within certain portions of HET. Then, at K = 6, KUN was delineated as a new genomic cluster. As K increased, DUO, HET, KUN, and QIR no longer exhibited obvious separation, while noticeable differentiation emerged among the three breeds POL, SUF, and TEX. This suggests that POL, SUF, and TEX may represent three hybrid varieties resulting from artificial selection processes.

### Detection of selection signatures

**Fig. 5:**
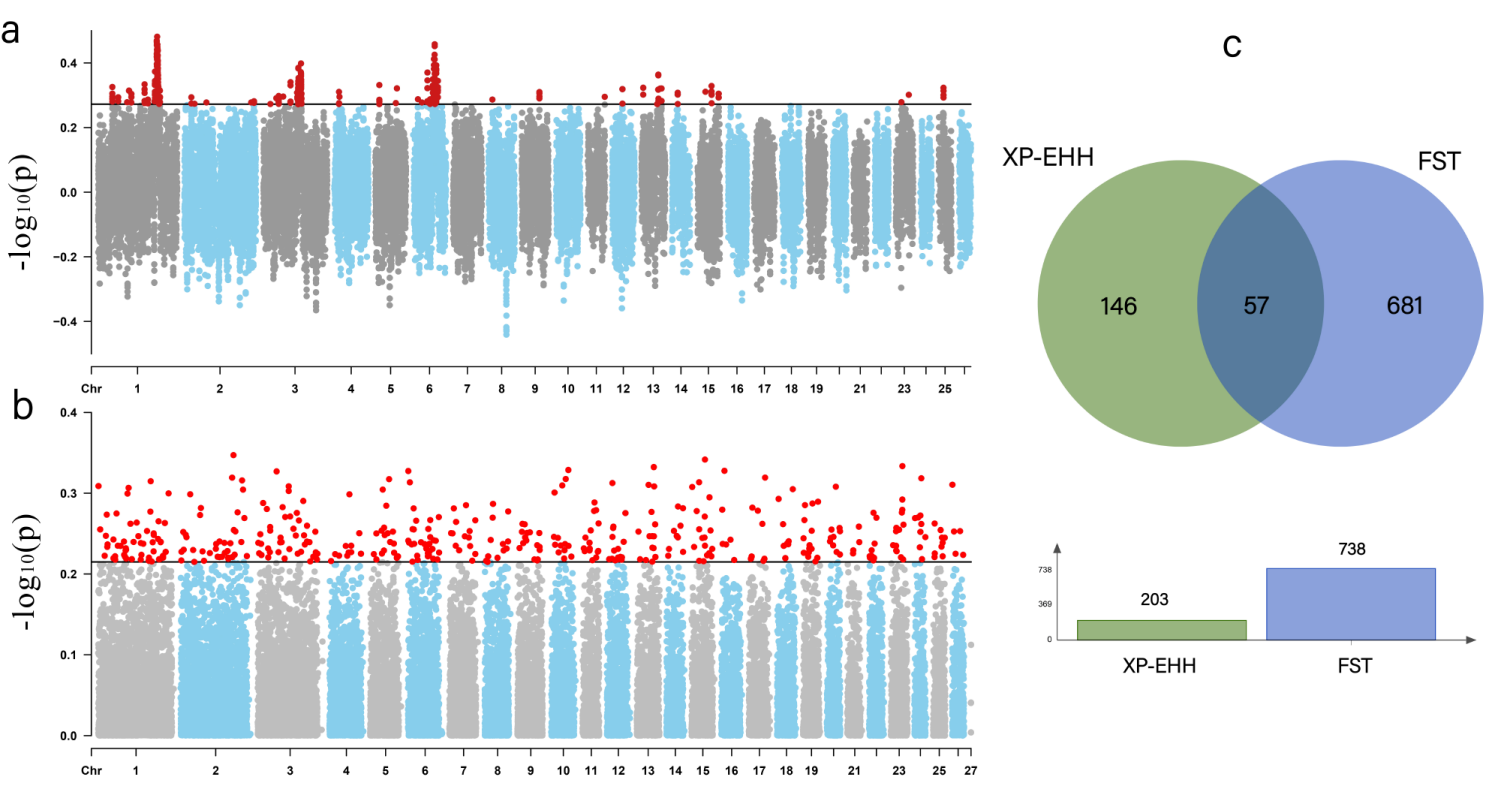
Manhattan plots of selection signatures determined by comparing the four Chinese local sheep breeds with three foreign sheep breeds using F_ST_ (**a**) and XP-EHH (**b**) methods. Wenn diagram(**c**) of XP-EHH intersection with Fst

The intersection and union relations of all selective scanning results obtained through the XP-EHH, FST, and iHS methods are presented in the Appendix Table (Table S1). In the XP-EHH analysis, a total of 203 candidate genes were identified across 15 autosomes at a threshold of 1% (Fig. 5a, Table S2). Subsequent GO enrichment analysis on these candidate genes revealed the top three significant GO terms: keratin filament (GO:0045095; P-Value = 9.22024E-06), anterior/posterior pattern specification (GO:0009952; P-Value = 2.86195E-05), and chromatin binding (GO:0003682; P-Value = 0.001262505) (Table S3). In the F_ST_ analysis, a total of 738 candidate genes were identified across 26 autosomes at a threshold of 1% (Fig. 5b, Table S4). Subsequent GO enrichment analysis on these candidate genes revealed the top three significant GO terms: transcription factor activity, sequence-specific DNA binding (GO: 0003700; P-Value = 0.000151821), regulation of cardiac muscle cell action potential (GO:0098901; P-Value = 0.000176044), and regulation of heart rate (GO:0002027; P-Value = 0.000330621) (Table S5). At the threshold of 1%, both XP-EHH and F_ST_ analyses detected 57 overlapping candidate genes (Fig. 5c, Table S1).

**Fig. 6:**
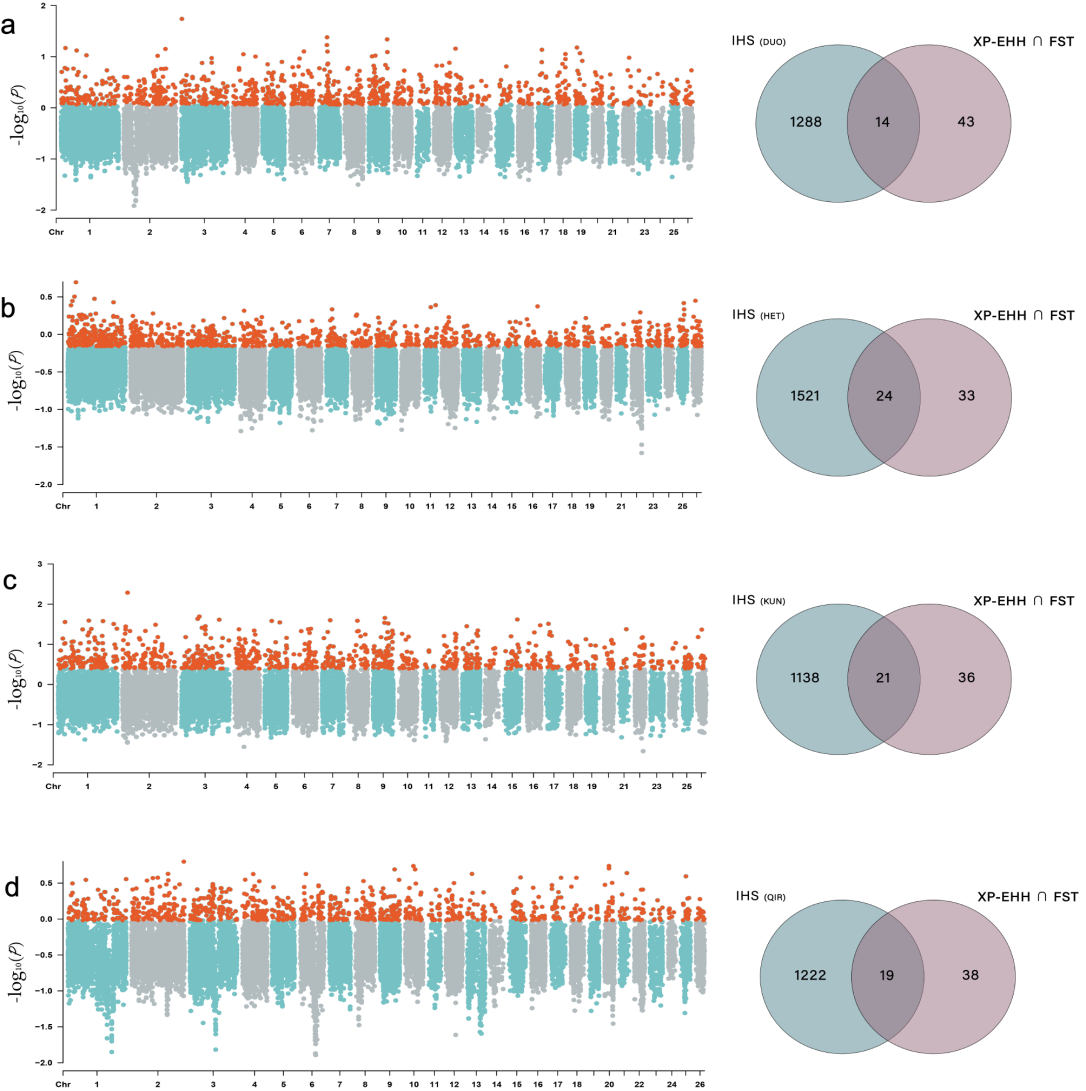
iHS Manhattan chart of DUO, HET, KUN and QIR. The intersection with F_ST_ and XP-EHH results obtained 14 (**a**), 24 (**b**), 21 (**c**) and 19 (**d**) genes, respectively

In the iHS test, we separately detected strongly selected regions in each local sheep breed, using a threshold of 5% (Table S6). Within the iHS-screened regions, we identified 1302, 1545, 1159 and 1241 candidate genes undergoing positive selection in the four indigenous sheep breeds (DUO, HET, KUN and QIR), respectively. By intersecting these results with those obtained from XP-EHH and F_ST_, we obtained 14, 24, 21 and 19 intersection genes, respectively (Fig. 6, Table S1).

**Fig. 7:**
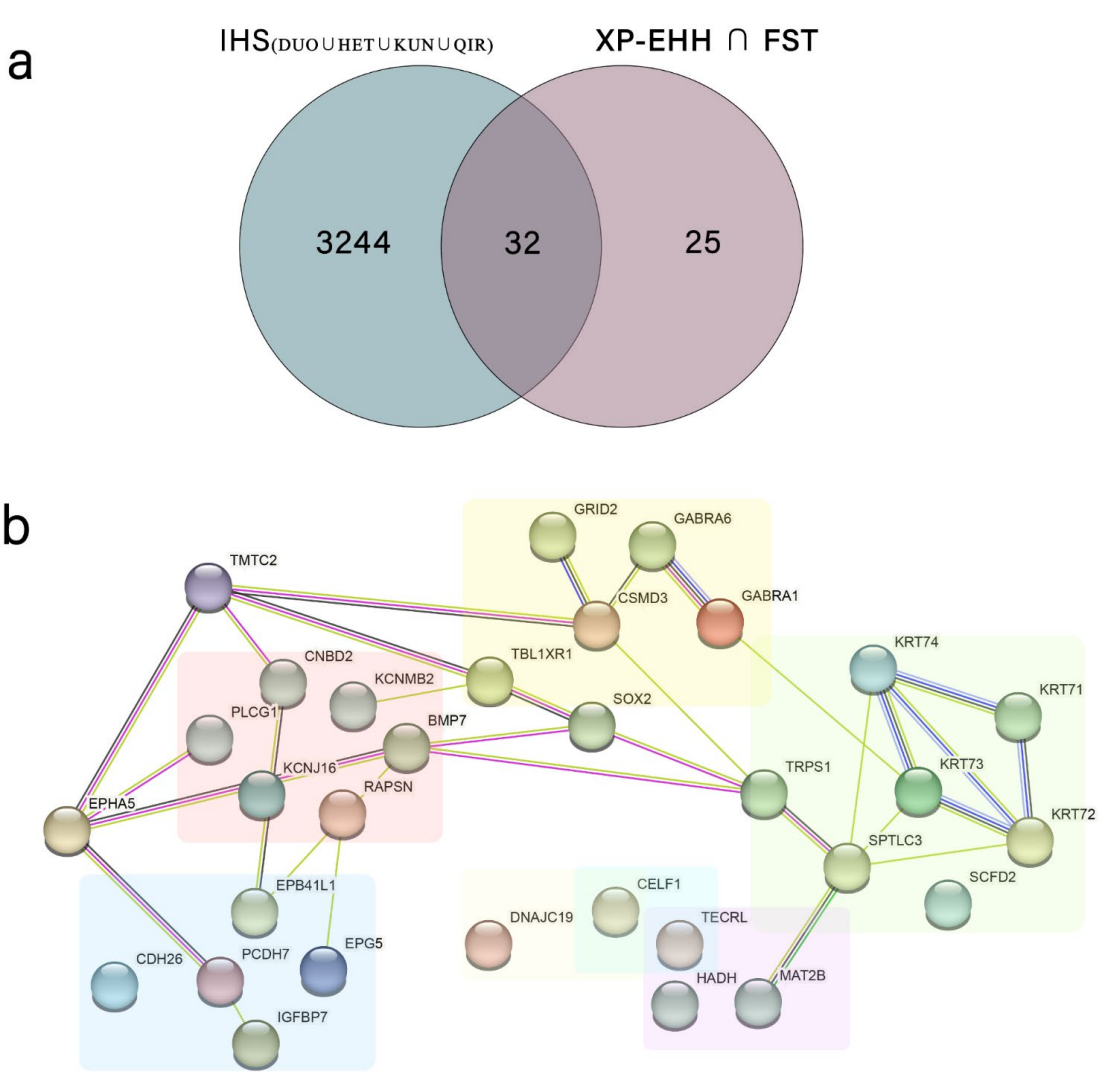
(**a**) 32 Overlapping genes subject to positive selection identified in four indigenous sheep breeds utilizing XP-EHH, F_ST_, and iHS Analyses (**b**) Gene networking analysis of 32 overlapping genes

Ultimately, using the three methods of detecting selection signals, XP-EHH, F_ST_ and iHS, we identified a total of 32 overlapping candidate genes across four indigenous sheep breeds inhabiting the southern edge of the Taklamakan Desert (Fig. 7a). These candidate genes are associated with wool follicle development and wool traits, adaptation to desert environments, reproductive traits, adaptation to high-altitude environment, and immune response in sheep (Table 2). Pathway enrichment analysis was performed on the candidate genes obtained by the intersection. (Table S7) Genes with similar functions within the overlapping candidate genes cluster aggregate and interact with each other (Fig. 7b).

**Table 2:** Identification of 32 overlapping genes subject to positive selection in four indigenous sheep breeds at the southern edge of the taklamakan desert utilizing XP-EHH, FST, and iHS analyses

Breeds	OAR	Gene position (bp)	Gene ID	Gene name	Function
HET	3	133608720-133618332	ENSOARG00000016914	*KRT73*	Wool fiber molding
HET	3	133651634-133659310	ENSOARG00000016946	*KRT74*	Wool fiber molding/Hair growth
HET	11	59517933-59519150	ENSOARG00000000206	*KCNJ16*	Growth and diastole of blood vessels
RIR	6	80712077-81089105	ENSOARG00000006407	*EPHA5*	Curly fleece trait
KUN	13	69403562-69417129	ENSOARG00000001700	*PLCG1*	Growth and diastole of blood vessels
HET KUN	1	203532281-203533162	ENSOARG00000014598	*SOX2*	Hair follicle development/Hair growth/Adaptability to extreme hypoxia and cold environments
HET KUN	1	206335541-206380437	ENSOARG00000020716	*KCNMB2*	Growth and diastole of blood vessels
HET KUN	3	118732543-118997463	ENSOARG00000015285	*TMTC2*	High altitude adaptability
HET KUN	5	70876729-70931718	ENSOARG00000013948	*GABRA1*	Motor coordination
HET KUN	5	70698294-70715186	ENSOARG00000013834	*GABRA6*	Motor coordination
HET KUN	9	61479981-61752577	ENSOARG00000010810	*TRPS1*	Hair follicle development/Wool traits
HET QIR	3	133668845-133676261	ENSOARG00000016956	*KRT71*	Wool fiber molding/ Curly fleece trait
HET QIR	3	133628051-133639981	ENSOARG00000016930	*KRT72*	Wool fiber moldings
HET QIR	15	75493445-75617600	ENSOARG00000006810	*RAPSN*	Body height
DUO QIR	6	17327261-17370182	ENSOARG00000008236	*HADH*	Lipid storage
KUN QIR	6	79660810-79793106	ENSOARG00000006132	*TECRL*	Pigment deposition / lipogenesis
KUN QIR	6	68512366-68900886	ENSOARG00000018700	*SCFD2*	Coat colour
KUN QIR	13	64673075-64723487	ENSOARG00000015308	*CNBD2*	Short stature
DUO KUN	13	55860624-55893440	ENSOARG00000015249	*CDH26*	leukocyte adhesion
DUO HET KUN	1	204290191-204294508	ENSOARG00000020670	*DNAJC19*	Mitochondria in heart muscle cells
DUO HET KUN	1	208006642-208180375	ENSOARG00000020719	*TBL1XR1*	Motor coordination
DUO HET QIR	5	72508398-72522813	ENSOARG00000014137	*MAT2B*	Lipid formation
DUO HET QIR	6	20565417-20685745	ENSOARG00020025493	*LEF-1*	Hair follicle development/Coat colour
DUO HET QIR	6	30768380-31534647	ENSOARG00000018241	*GRID2*	Litter size/motor and limb coordination/fat formation
DUO KUN QIR	13	64865115-64919869	ENSOARG00000015468	*EPB41L1*	Regulators of reproductive neuroendocrine
HET KUN QIR	13	6086778-6226790	ENSOARG00000010864	*SPTLC3*	Hair follicle development/Wool traits
DUO HET KUN QIR	6	49839231-49848437	ENSOARG00000009059	*PCDH7*	Resistance to parasitic infections
DUO HET KUN QIR	6	72423581-72437525	ENSOARG00000005761	*IGFBP7*	Ovarian follicular development
DUO HET KUN QIR	9	63608850-65007715	ENSOARG00000011267	*CSMD3*	Immune infiltration
DUO HET KUN QIR	13	58173131-58255337	ENSOARG00000018039	*BMP7*	Hair follicle development/Kidney development/Follicle development, follicle growth
DUO HET KUN QIR	15	75520222-75546682	ENSOARG00000006924	*CELF1*	Lens development/Sperm production/Heart function and morphology
DUO HET KUN QIR	23	45805752-45934686	ENSOARG00000002588	*EPG5*	Gonad formation and gametogenesis

## Discussion

### Population genetic diversity

Genetic variability serves as a crucial response mechanism of organisms adapting to environmental changes and stands as a pivotal aspect in biodiversity research [26]. Assessing genetic variation within species or population provides a theoretical bedrock for the preservation and enhancement of sheep germplasm resources [27].

MAF results revealed that the genetic variability in four indigenous sheep breeds from the southern edge of the Taklamakan Desert was comparable to that in other foreign sheep populations. Within our study cohorts, KUN exhibited the lowest F value (-0.0746), predominantly attributed to contemporary breeding strategies and the introduction of new individuals with diverse genetic backgrounds, effectively mitigating inbreeding. However, genetic diversity within the KUN remained relatively limited, primarily owing to their isolation in high-altitude mountainous regions and the small size of their core population. The implementation of rigorous scientific management practices is essential for conserving KUN ‘s genetic resources. Regular exchange of breeding rams among KUN could simultaneously enhance effective population size and enrich intraspecific genetic diversity [10].

It is notable that Ho closely approximated He across the four indigenous sheep breeds examined. This indicates that these populations are currently in a state of genetic equilibrium, neither undergoing significant selection pressure nor experiencing substantial inbreeding. Moreover, this finding indicates their substantial potential for selective breeding. In compared to DUO and KUN, HET and QIR exhibit slightly higher Ho than He. Considering their predominant distribution in the Hotan region of Xinjiang, a key junction along the Silk Road [28], we speculate that gene flow may significantly contribute to intraspecific genetic variation. This underscores the urgency of implementing genetic conservation programs aimed at preserving the unique genetic diversity of these breeds.

KUN demonstrated the lowest genetic variation, necessitating the implementation of a rational mating system, establishment of a gene bank, and promotion of gene flow among diverse populations as key strategies for conserving its genetic diversity. Conversely, HET and QIR showed high genetic variation, facilitating their adaptation to diverse environments and potential challenges. However, this diversity also complicates inter-population gene exchange. Future efforts for HET and QIR should focus on breeding individuals with superior traits through selective breeding or crossbreeding, while minimizing excessive artificial intervention.

The current study elucidated the genetic variation in indigenous sheep populations at the southern edge of the Taklamakan Desert through the analysis of genetic variation parameters within the populations. However, the small sample sizes of KUN and DUO may not sufficiently capture the genetic diversity within their populations. Additionally, selecting only adult ewes as samples may not fully reveal all the genetic characteristics within the populations. To enhance the representation of genetic diversity within the populations, future studies should expand the sample size of each sheep type and include male samples for analysis.

### Population genetic structure

Population genetic structure indicates a clear separation of indigenous sheep in Xinjiang from other sheep populations. The four indigenous sheep breeds in southern Xinjiang form two distinct groups with three sheep breeds from other countries, and the genetic resources of the four indigenous sheep breeds are closely related to each other. This clustering pattern is significantly associated with their geographical environment and genetic distance. Additionally, PCA and NJ tree analysis confirmed the presence of three distinct clusters within the HET, consistent with previous findings of Abied et al. [10]. on indigenous sheep breeds in China. Admixture analysis revealed that QIR displayed the highest proportion of Xinjiang sheep ancestry, while the genetic diversity in HET correlated with its geographic distribution [27]. HET living at intermediate and lower altitudes exhibited considerable genetic diversity, likely influenced by their geographic locations. Hetian, situated along the Silk Road, serves as a vital trade junction, possibly facilitating gene admixture in HET at intermediate and lower altitudes [29, 30]. The study also indicates that high-altitude HET have lower genetic diversity, primarily because they inhabit mountainous regions at higher altitudes. Geographical isolation is the main factor contributing to this lower genetic diversity in high-altitude HET populations [31, 32], implying a closer genetic relationship with their ancestral HET population.

### Selection signatures of candidate genes

To enhance the robustness of our results, we initially identified candidate genes with strong selection traits in local sheep by intersecting selection signals identified by the XP-EHH and F_ST_ methods. These candidate genes were than integrated with those identified through iHS analysis to detect alleles undergoing positive selection in each local sheep breed [21, 22]. Ultimately, the identified genes were associated with economic traits and adaptation to harsh environments.

#### Candidate genes associated with wool follicle development and wool traits

Wool, a key economic trait in wool-producing sheep, depends on the growth and development of hair follicles attached to the skin [33].

In this study, we identified five overlapping genes involved in hair follicle growth and development: *BMP7* [34, 35], *SOX2* [36, 37], *SPTLC3* [38], *LEF-1*, and *TRPS1*. *LEF-1*, a transcription factor, promotes the maturation of secondary hair follicles by regulating hair follicle development [39]. It has been reported that *SOX2* regulate hair growth by controlling BMP signaling [40]. *TRPS1* and *SPTLC3* are associated with cashmere wool characteristics [38].

Additionally, we also identified seven overlapping genes associated with wool traits. *KRT71*, *KRT72*, *KRT73*, and *KRT74* are specifically expressed in the inner root sheath of the hair follicles and play a vital role in the formation of wool fibers [41, 42]. Studies have shown a strong correlation between *KRT71* and curly hair phenotypes in sheep [43], dogs [44, 45], and cats [46]. *KRT72* is a major component of wool fibers [47], and *KRT74* is positively correlated with wool growth [48]. *EPHA5* and *SCFD2* are significantly associated with the curling characteristics and coat color of wool [49, 50].

#### Adaptive mechanism of the desert environment

The indigenous sheep breeds from the southern edge of the Taklimakan Desert are exposed to various biological and physical stressors throughout the year, such as high temperatures, drought, intense ultraviolet radiation, and parasites. Therefore, our candidate regions encompass several genes associated with adaptation to the desert environment and specific immune responses.

In arid or hot environments, many breeds typically exhibit smaller body sizes as an adaptation to cope with scarce water sources and temperature regulation [51]. This can explain the occurrence of genes such as *RAPSN*, *CNBD2*, *KCNJ16*, *KCNMB2*, *PLCG1*, and *BMP7* in the candidate regions. For instance, *RAPSN* is significant correlation with body mass in sheep [52], while *CNBD2* is associated with short stature, a trait also observed in human [53]. Additionally, *KCNJ16* [54], *KCNMB2* [55], and *PLCG1* [56] have been linked to vascular growth and dilation. Skin vascular dilation increases blood flow, facilitating heat dissipation, which explains the cooling mechanism in hot environments. *BMP7* is crucial for kidney function and development [51], as the kidney serves as a core adaptation for water retention and reabsorption in desert environments. Genes related to pigment deposition and eye lens development have been identified in the candidate regions. For instance, *CELF1* play vital roles in lens development [57], aiding in light refraction and filtering out ultraviolet rays. *TECRL* has a positive effect on pigment deposition [58]. These findings likely stem from natural selection resulting from prolonged exposure to intense sunlight and ultraviolet radiation.

We have identified *CSMD3* genes associated with cytokine signaling and immune infiltration [59], along with candidate genes involved in host defense, disease resistance, and inflammatory responses. For instance, *PCDH7* is a potential biomarker for host resistance to parasite infection [60], while *CDH26* regulates leukocyte activation and adhesion during allergic reactions [61].

#### Candidate genes associated to reproduction traits

Reproduction is an important trait in sheep breeding, influenced by various factors like environment, nutrition, and genetic factors. We have identified several candidate genes associated with reproductive traits, including those in gonadal development, gametogenesis, litter size, and embryonic development.

*IGFBP7* and *BMP7* are pivotal in reproductive processes, with *IGFBP7* [62]is highly expressed in ovaries, regulating follicular development and ovulation, while *BMP7* [63], a core BMP subfamily member, influences hormone production, granulosa cell development, and follicular development. *BMP7* is also a potential candidate gene for ovulation rate and litter size traits [64]. *EPG5* influences gonadal formation, gametogenesis and early embryonic differentiation [65, 66]. Targeted disruption of *CELF1* impairs spermatogenesis, causing male infertility [67]. *GRID2* is associated with litter size in Dazu Black goats [68]. *RBM38*, a target of the p53 family, affects embryonic development by modulating p53 accumulation via the Rbm38-p53 axis, particularly in a p21 (a conventional target of the p53 pathway) dependent manner at the morula/blastocyst stage [69–71].

*EPB41L1*/4.1 N, located within the hypothalamic-pituitary-gonadal (HPG) axis, regulates reproductive neuroendocrine function by facilitating the release of hormones (gonadotropin-releasing hormone/GnRH, growth hormone-releasing hormone/GHRH) in the hypothalamic-pituitary pathway [72]. GnRH acts on GnRH receptors (G-protein-coupled receptors) in the anterior pituitary, stimulating gonadotropin secretion (follicle-stimulating hormone/FSH, luteinizing hormone/LH, prolactin/PRL), thereby modulating reproductive system development.

#### Adaptive mechanism of the high-altitude environment

Animals inhabiting high-altitude areas face significant environmental challenges such as low oxygen, rugged terrain, and limited food availability. This study identified several genes associated with adaptability to high-altitude environments within the candidate regions.

*TMTC2* is a positively selected gene in high-altitude environments [73]. *SOX2* has been associated with extreme hypoxia and cold adaptation in Himalayan marmots [74].

Good motor coordination is crucial for sheep to navigate rugged high-altitude environments. *TBL1XR1*, associated with various developmental disorders affecting neural system functions [75], play a role in neural progenitor cell proliferation and differentiation. Loss or mutations in *TBL1XR1* can lead to abnormal motor coordination. *GABRA1* and *GABRA6*, exclusive to the nervous system, regulate brain development mechanisms [76, 77], and reduced levels may compromise motor function. *GRID2*, expressed in layer V cortical neurons involved in motor function formation, underpins motor control and limb coordination. Moreover, *GRID2* has been identified as a potential candidate gene for adipogenesis [78, 79].

In high-altitude conditions with low oxygen, sympathetic nervous system activation increases heart rate and cardiac output to enhance tissue oxygenation. We identified two candidate genes associated with cardiac function: *CELF1* deletion affects cardiac function and morphology in newborns, reducing cardiac vitality [80, 81]. *DNAJC19* (DnaJ heat shock protein family member C19), exhibits high expression in the heart’s mitochondria [82, 83], providing a significant amount of ATP for cardiac pumping function.

Adipose tissue serves as a crucial energy reserve for animals in extreme environments and during food scarcity, offering an adaptive response. We identified genes linked to sheep fat deposition: *MAT2B* promotes fat synthesis by regulating cellular levels of S-adenosylmethionine (SAMe) [84]. TECRL participates in lipid production reactions [85]. *HADH* participates in β-oxidation and correlates with lipid content and droplet numbers in cells [86]; its knockout inhibits lipid storage.

This study utilized diverse selection signal scanning methods to reveal the distinctive evolutionary trajectories of indigenous sheep from the southern edge of the Taklamakan Desert. It identified candidate genes in the genome associated with wool follicle development, wool traits, environmental adaptability, disease resistance, reproduction, and high-altitude adaptation. These findings provide new germplasm resources and breeding strategies for sheep breeding and improvement, offering important references for other mammals in devising climate change coping strategies. In the future, we plan to conduct functional validation of these adaptive genes and further elucidate the molecular mechanisms underlying the adaptive evolution of sheep by integrating multiple omics data. Although this study detected genetic variations in four indigenous sheep breeds using high-throughput sequencing technology, limitations in genetic marker coverage may have hindered the comprehensive identification of all key genetic variations. However, with the advancement of high-throughput sequencing technology, our aim is to enhance sequencing depth, broaden the range of breeds, and augment population sizes comprehensively reveal the genetic diversity and evolutionary history of sheep populations.

## Conclusions

The study revealed the genetic variability of native sheep breeds from the southern edge of the Taklimakan Desert, which provides new insights for the conservation of population in the future. Additionally, based on geographical and genetic relationships, the four native sheep breeds from the southern edge of the Taklimakan Desert and three sheep breeds from other countries were clustered into two groups, and HET based on geographical location were made further subdivision into three clusters using. Analysis of selection characteristics made a certain for various candidate genomic regions, which were associated with wool follicle development and wool traits, desert environmental adaptability, disease resistance, reproduction, and high-altitude adaptation. At the molecular level, we elucidated the genetic mechanisms underlying the adaptability of native sheep breeds from the southern edge of the Taklimakan Desert in the extreme environments. This provides new insights into the conservation and sustainable utilization of genetic resources in native sheep and offering important references for sheep and other mammals in devising climate change coping strategies.

### Electronic supplementary material

Below is the link to the electronic supplementary material.

Supplementary Material 1

Supplementary Material 2

Supplementary Material 3

Supplementary Material 4

Supplementary Material 5

Supplementary Material 6

Supplementary Material 7

## Data Availability

The datasets analysed during the current study are available in the OMIX, China National Center for Bioinformation / Beijing Institute of Genomics, Chinese Academy of Sciences repository, (https://ngdc.cncb.ac.cn/omix; GSA: OMIX006742).

## Footnotes

1 http://www.sheephapmap.org.

2 https://github.com/hewm2008/VCF2Dis.

3 http://www.atgc-montpellier.fr/fastme/execution.php.

4 https://itol.embl.de/itol.cgi.

5 https://www.ncbi.nlm.nih.gov/pmc/.

6 http://www.ncbi.nlm.nih.gov/omim.

7 https://david.ncifcrf.gov/.

8 https://www.ebi.ac.uk/QuickGO/.

9 https://www.genome.jp/kegg/kegg2.html.

## Declarations

### Benefit sharing

The authors declare no benefit sharing.
